# The Patent Foramen Ovale and Migraine: Associated Mechanisms and Perspectives from MRI Evidence

**DOI:** 10.3390/brainsci12070941

**Published:** 2022-07-18

**Authors:** Wenfei Cao, Yinbo Shen, Jiaqi Zhong, Zhenhong Chen, Nizhuan Wang, Jiajun Yang

**Affiliations:** 1 Department of Neurology, Shanghai Jiao Tong University Affiliated Sixth People’s Hospital, Shanghai 200233, China; wallacecaowenfei@163.com (W.C.); syb19970408@163.com (Y.S.); zhongjiaqi1015@163.com (J.Z.); zhenhong0512@163.com (Z.C.); 2 School of Biomedical Engineering, ShanghaiTech University, Shanghai 201210, China

**Keywords:** patent foramen ovale, right-to-left shunt, migraine, mechanism, gray matter, white matter hyperintensities, magnetic resonance imaging, functional magnetic resonance imaging

## Abstract

Migraine is a common neurological disease with a still-unclear etiology and pathogenesis. Patent foramen ovale (PFO) is a kind of congenital heart disease that leads to a right-to-left shunt (RLS). Although previous studies have shown that PFO has an effect on migraine, a clear conclusion about the link between PFO and migraine is lacking. We first summarized the PFO potential mechanisms associated with migraine, including microembolus-triggered cortical spreading depression (CSD), the vasoactive substance hypothesis, impaired cerebral autoregulation (CA), and a common genetic basis. Further, we analyzed the changes in brain structure and function in migraine patients and migraine patients with PFO. We found that in migraine patients with PFO, the presence of PFO may affect the structure of the cerebral cortex and the integrity of white matter, which is mainly locked in subcortical, deep white matter, and posterior circulation, and may lead to changes in brain function, such as cerebellum and colliculus, which are involved in the processing and transmission of pain. In summary, this paper provides neuroimaging evidence and new insights into the correlation between PFO and migraine, which will help to clarify the etiology and pathogenesis of migraine, and aid in the diagnosis and treatment of migraine in the future.

## 1. Introduction

Migraine is a common chronic disease of the nervous system [1]. A recent study in 2022 reported that the number of migraine patients worldwide reached 1.1 billion in 2019 [2]. Interestingly, the diagnostic criteria for migraine are mainly based on the third edition of the International Classification of Headache Disorders (ICHD-3). It is typically characterized by a unilateral, moderate-to-severe, recurrent, and pulsatile headache, lasting for 4–72 h, and can be accompanied by nausea, vomiting, photophobia, phonophobia, and other symptoms [3]. The proportion of female migraine patients is higher than that of men, and it is also the main cause of disability among people under the age of 50, especially women [4]. In 2019, the United States released a population report on migraine, showing that it has a negative impact on marriage, childcare, love, family relations, career, economy, and physical and mental health [5]. A Global Burden of Diseases (GBD) study in 2019 showed that migraine was the fifth cause of disability-adjusted life-years (DALYs) among people aged 25–49, who had the greatest impact on productive forces [6]. Thus, migraine not only affects personal health and life but also has a negative impact on the global economy and productivity of a society. However, the pathophysiology of migraine has not been fully elucidated, and the trigeminovascular system is the main anatomical and physiological basis for exploring the mechanism of migraine [7]. Specifically, trigeminal ganglion neurons receive nociceptive impulses from meninges and their blood vessels and then convey them to second-order trigeminovascular neurons, including the spinal trigeminal nucleus, which activates and sensitizes multiple nuclei in the brainstem and thalamus. The third-order trigeminovascular neurons in the thalamus project to cortical areas, including the somatosensory cortex, visual cortex, and other cortical areas, ultimately resulting in migraine headaches [7]. CSD is the main theory explaining the aura of migraine [7]. In recent years, it was found that the prevalence of PFO in migraine patients is significantly higher than that in the healthy population, and a growing number of studies focus on exploring the role of PFO in the pathophysiology of migraine.

The foramen ovale is a physiological channel located on the septum between the left and right atrium during the fetal period, which connects the left and right atrium [8]. It is usually closed after birth, but if the channel is not fully closed after the age of three, it is called PFO. When the right atrial pressure is higher than the left, patients with PFO show RLS [9]. The causes of RLS include pulmonary arteriovenous malformation, atrial septal defect, and PFO, of which PFO is the most common, accounting for 95% of all RLS [10]. A recent expert guide shows that the incidence of PFO is 30% in people aged 1–29, 25% in people aged 30–79, and 20.2% in those over 80 [11]. It is generally believed that the incidence of PFO is about 25% at the population level [11]. Imaging examinations are commonly used in the clinical diagnosis of PFO, including transthoracic echocardiography (TTE), contrast transthoracic echocardiography (cTTE), transesophageal echocardiography (TEE), contrast transesophageal echocardiography (cTEE), contrast-enhanced transcranial doppler (cTCD) and cardiac catheterization [11]. A meta-analysis in 2014 showed that the sensitivity and specificity of cTCD were 97% and 93%, respectively [12]. Furthermore, cTCD can be used to diagnose cardiac shunt according to the time of microbubbles (MB) appearance [13]. Clinically, in some centers, patients suspected of PFO are generally screened for cTCD because of its high sensitivity [14]. Notably, TTE and TEE, which can observe the anatomical structure of the heart, clearly show whether there is PFO and the size of PFO [15]. Both cTTE and cTEE can determine whether there is an RLS and the amount of RLS. TEE is a semi-invasive examination with a sensitivity of about 90%, but there is still a high misdiagnosis rate of 10% [16]. Compared with TTE, TEE is not disturbed by pulmonary gas and is the gold standard for the diagnosis of PFO [17]. Cardiac catheterization, which is quite expensive and invasive, is the most accurate method used to confirm PFO [14]. However, it is rarely used in PFO screenings in clinics.

## 2. PFO and Migraine

**Figure 1:**
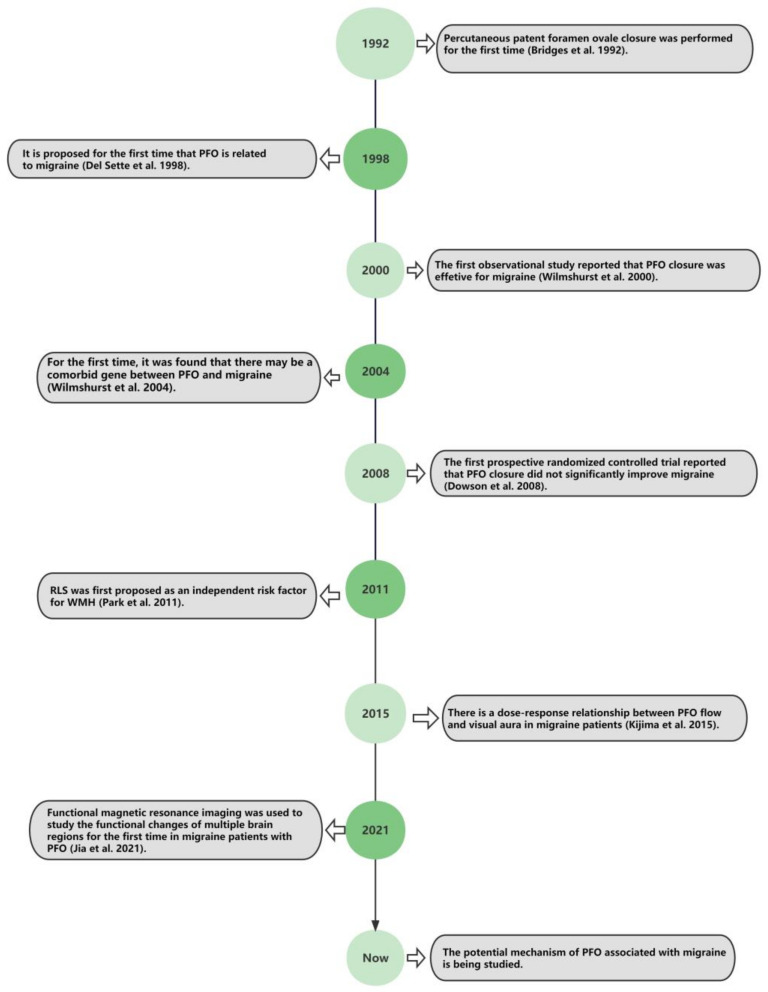
Timeline of important events related to PFO and migraine [18,20,25,26,27,28,29,30].

A series of important events are related to PFO and migraine, which are depicted in Figure 1. In 1998, Del et al. first revealed that PFO was an independent risk factor for migraine through a case-control study, and the prevalence of PFO in migraine patients with aura was significantly higher than that in healthy controls [18]. Since then, more and more researchers have explored the relationship between PFO and migraine and further studied the intervention effect of PFO closure on migraine. A large number of observational studies have shown that the prevalence of PFO in people with migraine aura is higher than that in the general population [19]. Interestingly, in 2015, Kijima et al. found that there was a positive correlation between the flow of PFO and the frequency of visual aura [20]. Furthermore, the presence of RLS is related to the onset age of migraine with aura [21]. The greater the severity of RLS, the earlier the onset is. In particular, if RLS is combined with atrial septal aneurysms, the onset age is further reduced [21]. The meta-analysis [22] in 2019 showed that the incidence of PFO in migraine was 3.19 times higher than that in healthy controls, and the incidence of PFO in migraine patients with aura was 2.32 times higher than that in migraine patients without aura. This suggests that PFO is significantly correlated with migraine, especially migraine with aura. In a prospective study, Zhao et al. described that the prevalence of large RLS, large-size PFO (≥2.0 mm), and permanent RLS in migraine patients with aura was higher than that in healthy controls [23]. Meanwhile, Tang et al. [24] recently found that the prevalence of migraine without aura in the PFO group was significantly higher than that in the group without PFO, so PFO was also closely related to migraine without aura.

In order to explore the potential mechanism of the correlation between PFO and migraine, researchers are committed to studying the pathophysiology of both PFO and migraine. In 2004, the genetic mechanism was first found to be involved [25], which attracted the attention of scholars. In addition, with the wide application of brain imaging technology, neurologists try to use neuroimaging to find the structural and functional changes in the brain in migraine patients with PFO [26,27], and we introduced this in detail below.

PFO closure was first implemented in 1992 [28], followed by an observational experiment in 2000, which first reported that PFO closure effectively relieved the symptoms of migraine patients [29]. Although Dowson et al. designed a prospective, randomized, clinical controlled trial in 2008, the results revealed that PFO closure did not reduce migraine attacks [30]. However, many scholars criticized the experiment and pointed out its bias and shortcomings. In the following years, some scholars conducted randomized controlled clinical trials to verify whether PFO closure is beneficial to migraine patients. Notably, in 2021, Mojadidi et al. [31] summarized all experimental data from two randomized clinical trials on PFO occlusion: a premium trial [32] and a Prima trial [33]. The corresponding results showed that PFO closure is beneficial and can significantly reduce the number of migraine days per month and the frequency of attacks per month [31]. Moreover, it is possible to completely cure some migraine patients [31]. The above reveals the inseparable relationship between PFO and migraine.

However, the mechanism by which PFO affects migraine has not been fully clarified. There are some hypotheses that aim to explain the pathogenesis of PFO and migraine. Firstly, it is believed that microembolus can trigger CSD, which is the most likely hypothesis regarding the pathophysiological mechanism of migraine that is associated with PFO. CSD is considered to be the pathological mechanism of the migraine aura, which can activate the trigeminal neurovascular system and cause headaches [7]. Specifically, CSD, also known as diffusion depolarization, is a depolarization wave of neurons and glial cells, which spreads slowly in the gray matter on the surface of the brain [34]. Interestingly, CSD is essentially an excitatory wave propagating at the speed of 2–6 mm/min; however, due to the disruption in ion gradients inside and outside the cell caused by continuous depolarization, the action potential of neurons cannot be fired, and then, there is the phenomenon of inhibition [35]. Previous studies have found multiple CSD in patients with focal ischemia [36], so the brain injury caused by ischemia may induce CSD. Notably, animal experiments also confirmed this phenomenon, where Nozari et al. [37] established an animal model of CSD, which was induced by microembolization to explore the mechanism of migraine caused by PFO. It was found that air microembolization caused transient hypo-perfusion, which triggered CSD [37]. Furthermore, the spread of CSD can open the neuronal pannexin-1 channel, leading to the release of inflammatory factors such as prostaglandins, nitric oxide, etc., which can trigger the trigeminal neurovascular system to produce pain of migraine with aura [1]. Therefore, it can be understood that the existence of PFO causes the abnormal microembolism of the venous system to directly enter the arterial circulation and temporarily affect the arterial microcirculation, resulting in hypoperfusion and brain injury that can trigger CSD and lead to migraine. This may also explain that PFO is more common in migraine patients with aura. Interestingly, the recent literature supported the idea that microembolic signals are independent predictors of higher cortical dysfunction in migraine patients with aura [38]. In addition, because RLS reduces the blood oxygen saturation in cerebral artery blood, it also triggers CSD [39].

Another potential mechanism is the vasoactive substance hypothesis. In 2006, Wilmshurst et al. [40] first proposed the hypothesis that cardiac and pulmonary pathology may play a role in the mechanism of migraine, suggesting that due to the existence of RLS in the heart, some venous factors, including 5-hydroxytryptamine (5HT), bypass pulmonary metabolism and enter the arterial blood to participate in the migraine mechanism. At present, the hypothesis is that vasoactive agents from the venous system, such as 5-HT, nitric oxide, kinin, and endothelin, bypass the metabolism of the pulmonary circulation and directly enter the arterial system. Notably, 5-HT, which is an important neuromodulator related to pain disorders, plays a role by binding to a variety of receptors [41]. This not only senses pain but also has an analgesic effect, which mainly depends on the subtype of the receptor [42]. On the one hand, the sheer force caused by PFO can activate platelets and release 5-HT [42]. On the other hand, the presence of PFO cause 5-HT to bypass the degradation of pulmonary monoamine oxidase [40]. Ultimately, a large amount of 5-HT arrives and activates pain fibers around meningeal vessels through blood circulation [43]. In addition, the pain effect of serotonin is accompanied by the release of pain mediators such as NO, PGI2, and CGRP, which cause vasodilation and further activate and sensitize trigeminal sensory neurons [43]. This hypothesis was supported by clinical experiments. For example, findings reported in 2016 evaluated the changes in vasoactive substances CGRP, 5-HT, NO, and substance P in migraine patients before and after the percutaneous occlusion of PFO, suggesting that migraine symptoms of patients were significantly improved, and the contents of four vasoactive substances in peripheral blood were reduced after closure [44].

Thirdly, some studies also implied that impaired CA might also be involved in the pathophysiological process of PFO-related migraine. CA means that in the face of a wide range of blood pressure fluctuations, brain perfusion can be maintained at a relatively stable level by automatically regulating the contraction and expansion of blood vessels [45]. Moreover, if CA is normal, the blood flow changes caused by PFO are regulated in a stable state rather than hypo-perfusion. Interestingly, Guo et al. [46] confirmed that compared with migraine patients without RLS, patients with RLS had significantly lower cerebral blood flow velocity and autoregulation index, suggesting that the CA in migraine patients with RLS was impaired.

Finally, heredity may also play an important role in the pathophysiology of PFO comorbid migraine. As with congenital heart disease, the etiology of PFO is related to environmental and genetic factors. Mutations in genes that encode structural proteins and transcription factors (which are involved in heart development) may play a role in the genetic processes of some PFOs [8]. For example, in 2004, Wilmshurst et al. [25] first proposed that PFO and migraine may share a common genetic basis. Subsequently, a 2010 meta-analysis [47], which looked at whether C677T polymorphism in the MTHFR gene and the I/D polymorphism in the ACE gene was associated with migraine, showed that the MTHFR 677TT genotype was correlated with an increased risk of migraine with aura. Notably, Szczygioł et al. [48] further revealed a significant increase in the prevalence of PFO in migraine patients with aura with a homozygous recessive genotype of MTHFR, suggesting that PFO and migraine may share a common genetic basis.

In conclusion, a large amount of the above evidence suggests that PFO is closely related to migraine in terms of its microembolus, vasoactive substance, impaired CA, and heredity features.

## 3. MRI and Migraine

Recently, advanced MRI techniques have become a useful technology for studying migraine biomarkers, which can identify the microstructural damage in the brains of migraine patients to provide a reliable basis for clinical diagnosis. A large number of studies have used MRI to explore the pathophysiological mechanisms of migraine, including structural MRI and functional magnetic resonance imaging (fMRI) (see Table 1).

**Table 1:** Summary of main MRI investigations of migraine.

Technique	Study	Population	Target Location	Findings	Reference
VBM	Valfrè et al.	CM	Right superior temporal gyrus, right inferior frontal gyrus, and left precentral gyrus	The significant reduction in gray matter in several cortical areas involved in the pain circuit is related to migraine.	[49]
	Palm-Meinders et al.	MA and MwoA	Right occipital cortex	Migraine patients show small changes in brain volume in cortical areas involving visual motor processing.	[50]
	Chen et al.	EM	Periaqueductal gray (PAG)	PAG volume expansion proves the existence of brain injury and can be used as an imaging biomarker for the diagnosis and evaluation of migraine.	[51]
	Zhang et al.	MwoA	Bilateral cerebellar culmen, occipital–temporal cortex, right insula, left postcentral gyrus, superior parietal lobule, right lateral occipital cortex, left rostral middle frontal gyrus	The significant changes in these gray areas may be related to the perception, integration, and processing of pain.	[52]
	Qin et al.	MwoA	Cerebellum and brainstem	The microstructure changes in the cerebellum and local brainstem that appeared in MwoA indicate that they are involved in the pathologies of migraine without aura.	[53]
	Neeb et al.	CM and EM	Amygdala and putamen, frontal and temporal gyrus, left cuneus	GM changes are associated with migraine frequency, so the increase in gray matter volume may reflect the remodeling of the central nervous system.	[54]
SBM	Schwedt et al.	EM and CM	Temporal pole, anterior cingulate cortex, superior temporal lobe, entorhinal cortex, medial orbital frontal gyrus, and pars triangularis	Compared with EM, the cortical surface area, cortical thickness, and cortical volume in CM showed significant differences, and these differences can be used to accurately distinguish CM and EM.	[55]
DTI	Planchuelo-Gómez et al.	EM and CM	Bilateral external capsule	Compared with patients with episodic migraine, patients with chronic migraine may have axonal integrity damage in the first few months of chronic migraine attacks.	[56]
	Porcaro et al.	MwoA	Hypothalamic	The hypothalamus plays a crucial role in the onset of migraine.	[57]
	Tantik Pak et al.	Migraine patients	Corpus callosum	The corpus callosum of migraine patients showed microstructural changes.	[58]
Task-fMRI	Cao et al.	MA and MwoA	Visual stimuli, red nucleus and substantia nigra	The brain stem is activated during migraine attacks.	[59]
	Yu et al.	VM and MwoA	Vestibular stimulation, parietal lobe, temporal lobe, insular lobe, cingulate gyrus, thalamus, caudate nucleus, optic radiation, precuneus.	The abnormal activation of the thalamus and fusiform gyrus may be involved in the pathophysiological mechanism of VM.	[60]
	Stankewitz et al.	Migraine patients	Olfactory stimulation, brain areas, rostral pons	The increased activity in the rostral part of pons indicates that there may be a physiological relationship between olfaction and the trigeminal nociceptive pathway.	[61]
RS-fMRI	Cui et al.	MwoA	Vision-related brain networks	Visual–related brain networks are dysfunctional in migraine patients.	[62]
	Tu et al.	MwoA	The occipital lobe, the sensorimotor network, part of the medial-cerebellum, the cingulo–opercular network, the default mode network, the frontal–parietal network	The functional connections of 6 regions in patients with migraine without aura have specific changes, which can be used to distinguish migraine patients from healthy controls.	[63]

EM: episodic migraine; CM: chronic migraine; MA: migraine with aura; CC: corpus callosum; VM: vestibular migraine, MwoA: migraine without aura; VBM: voxel-based morphometry; SBM: surface-based morphometry; DTI: diffusion tensor imaging; RS-fMRI: resting-state fMRI.

Some studies found that there are changes in gray matter volume in migraine patients, confirming the trigeminovascular system hypothesis, which states that the cortex is involved in processing the stimulating signals from neurons to produce pain [7]. In migraine patients, a significant reduction in gray matter in several of the cortical areas was observed to be involved in pain circuitry, such as the right superior temporal gyrus, right inferior frontal gyrus, and left precentral gyrus [49]; decreased gray matter volume was observed in the visual areas V3 and V5 of the right occipital cortex [50]; decreased volume of the spinal trigeminal nucleus (SPV) was involved in transmitting and regulating traumatic information of intracranial vessels and meninges, and the decreased volume of the cerebellum was related to pain information [51]. Overall, gray matter reduction reflects tissue atrophy, and repeated ischemia caused by abnormal cerebral blood flow may lead to gray matter changes [49]. Moreover, an increase in gray matter volume can be observed in migraine patients, which may be due to repeated chronic pain leading to the remodeling of the central nervous system [54]. Migraine patients are not only accompanied by changes in the gray matter but also accompanied by white matter destruction, such as the reduction in the fractional anisotropy (FA) of the external capsule and corpus callosum [56,58], and migraine patients are more likely to show white matter hyperintensities (WMH) than healthy controls [64], although the distribution of WMH is not related to the type of aura in migraine patients with aura [65]. This means that migraine patients may suffer from microvascular damage, resulting in the destruction of the integrity of axons.

In addition to the structural changes in migraine, we need to pay attention to the functional changes detected by fMRI. On the one hand, the activation of the thalamus, brainstem, and related functional areas such as the cingulate gyrus, insular lobe, and temporal lobe can be observed under task stimulation, indicating that these areas are involved in the pathophysiological process of inducing migraine [57,58,59]. On the other hand, abnormal brain networks and functional connections can also be observed in the resting state, including the occipital lobe, the sensorimotor network, bilateral lateral and inferior cerebellum, the cingulo–opercular network, the default mode network, and the frontal–parietal network [62,63]. In recent years, research on the small-world network of migraine patients and the study of dynamic functional connectome patterns both support the idea that the thalamus, occipital lobe, and basal nucleus play a vital role in the process of relaying pain, regulating vision, and integrating pain [66,67].

Similarly, the existence of PFO can lead to cerebral ischemia and microcirculation disorders, and scholars studied whether there are similar or specific structural and functional changes in migraine patients with PFO, which are introduced below.

## 4. MRI Evidence in Migraine Patients with PFO

### 4.1. Structural MRI-Based Evidence

#### 4.1.1. Gray Matter Changes

Voxel-based morphometry (VBM), which can identify the changes in brain microstructure such as gray matter volume and density and cortical thickness, is a commonly used technique to study gray matter (GM). Although there is no direct study of gray matter changes in migraine patients with PFO using VBM, there may be indirect evidence proving that PFO is related to migraine. Many studies have used VBM to study gray matter changes in migraine patients. In order to explore whether there is a consistent correlation between migraine and gray matter anomalies, Dai et al. [68] included VBM studies of migraine published from 2000 to 2014 and conducted a quantitative mate analysis for the first time. Interestingly, the results suggested that migraine patients showed a decrease in GM volume, proving that abnormal gray matter was involved in the neural network of pain processing [68].

Most notably, Kang et al. [69] observed the imaging characteristics of a migraine patient with PFO during the attack period and the asymptomatic period after the attack. Reversible imaging changes were found in the fluid-attenuated inversion recovery (FLAIR) image of brain MRI. During persistent migraine attacks, the sulcal hyperintensities on the FLAIR image were found in the frontoparietal cortex, but there was no obvious abnormal signal in the FLAIR image 3 days after the migraine symptoms disappeared [69]. Although this is only a special case report, it may confirm that the microemboli produced by RLS in PFO patients can lead to disturbances in intracranial arterial microcirculation, which triggers the onset of migraine. When the microcirculation disturbance was relieved, the migraine symptoms were also alleviated, which may be related to the size and duration of the microemboli.

In conclusion, the above evidence supports the idea that PFO and migraine may have a shared mechanism, but more studies on gray matter manifestations in migraine patients with PFO are needed in the future.

#### 4.1.2. White Matter Changes

The changes in white matter in migraine patients with PFO aroused the interest of scholars. At present, most research is about WMH related to migraine with PFO. There is evidence that PFO is associated with white matter hyperintensity in migraine patients. Table 2 lists these findings and the distribution area of WMH in migraine patients with PFO.

**Table 2:** Evidence from structural MRI between PFO and WMH in migraine patients.

Literature	Patients	Location of WMH	Findings	Reference
Signoriello et al.	Migraine patients	Occipital lesions and juxtacortical seat.	PFO may be associated with white matter lesions in migraine patients, especially those with occipital lesions and visual aura.	[70]
Park et al.	Tension-type headache and migraineurs	Deep white matter	In young migraine patients, small deep WMH is associated with RLS.	[26]
Yoon et al.	Migraine patients	Juxtacortex and cortico–subcortical junction.	Juxtacortical spots on FLAIR images may be related to the presence of PFO in migraine patients.	[71]
Iwasaki et al.	MA and MwoA	Deep or subcortical white matters	RLS may be associated with WMH in Japanese migraine patients.	[72]

MA: migraine with aura; MwoA: migraine without aura; WMH: white matter hyperintensities; FLAIR: fluid-attenuated inversion recovery; PFO: patent foramen ovale; RLS: right-to-left shunt.

Interestingly, a previous study [73] showed that the sympathetic nerves of the anterior and posterior circulation regulate blood flow in different ways. Hayashida et al. [74] also found that the distribution of embolism in PFO mainly accumulated in the posterior circulation through a study tracking the distribution of abnormal embolism from PFO in the brain. This is further confirmed by a recent study in which Signoriello et al. [70] found that the location of hyperintensities on FLAIR image in migraine patients with PFO was more concentrated in the posterior circulation, especially the occipital lobe. This indicates that PFO may be related to the localization of WMH. Another finding supports the involvement of PFO in WMH. For example, Xie et al. [75] compared the breath-holding index (BHI) of the middle cerebral artery in migraine patients with and without RLS to explore whether RLS affects cerebrovascular reactivity (CVR), which can be evaluated by BHI. They further evaluated whether the change in vascular reactivity is a potential mechanism of WMH. The results suggest that BHI decreases in RLS patients, and the decrease in CVR is independently related to the occurrence of WMH [75].

Furthermore, scholars studied whether the grading of RLS has an impact on WMH. RLS can be classified according to the number of microbubbles that are detected by cTCD. If there are no microbubbles, this is classified as grade 0; if there are 1–10 microbubbles (one side), it is classified as grade I, also known as small RLS; if there are more than 10 microbubbles (one side), but a curtain pattern is not formed, it is classified as grade II, also known as medium RLS; if there are more than 10 microbubbles (one side) with curtain patterns, it is classified as grade III, also known as large RLS [11]. Yoon et al. [71] conducted a retrospective case-control study, including 49 migraine patients and 49 healthy controls, and RLS was divided into four levels according to cTCD. The results were that with the increase in RLS severity, the area of paracortical hyperintensities also increased [71]. In addition, a study [76] revealed that the large RLS might be a risk factor for WMH in migraine patients. However, there is little evidence that RLS grading affects WMH. Several studies have found that RLS classification has no significant correlation with the number and the total volume of WMH [77,78,79]. Obviously, due to the unclear mechanism, we cannot provide enough evidence to prove that RLS grading has an impact on WMH.

In summary, although no definite conclusion that PFO is specifically related to WMH in migraine patients was reached, more convincing evidence should be investigated to determine their correlation in the future.

### 4.2. Functional MRI-Based Evidence

Functional magnetic resonance imaging (fMRI) is an imaging technique used to study neural function. The most commonly used technique is the blood-oxygen-level-dependent (BOLD) fMRI technique to measure the blood-oxygen-level-dependent signal, which includes two main categories: to study the task state, that is, the activation state of brain regions under some stimuli, which is called task-fMRI; to study the functional connectivity of the brain in resting state, which is called RS-fMRI [80]. In fact, fMRI was widely used in studies on migraine, but there are few studies focusing on migraine with PFO comorbidity. Recently, Jia et al. [27] used RS-fMRI to evaluate the functional changes in multiple brain regions in migraine patients. It was observed that patients with PFO had different manifestations in the temporal lobe, bilateral cerebellar hemisphere, and thalamus (all three have been proved to be involved in the pathophysiological mechanisms of migraine) compared with patients without PFO, and patients with PFO have a more prominent cognitive impairment, such as visual space and execution, orientation and attention [27]. Although this study has certain limitations and the existence of research cannot be ruled out, it is undeniable that it provides a new way of exploring the elusive migraine in the future. Therefore, we need more research on fMRI in migraine patients with PFO to reveal whether PFO is involved in the changes in brain function.

## 5. Discussion

Migraine is a disabling chronic disease of the nervous system, which has a serious negative impact on the quality of life and spirit of patients. The correlation between PFO and migraine and PFO treatments has always been controversial and is also a research hotspot. An in-depth understanding of the relationship between PFO and migraine will provide important guidance for the screening and management of PFO. In this context, we summarized the development of the possible pathophysiological mechanisms regarding PFO and migraine and further summarized the neuroimaging and abnormal manifestations of migraine patients. Most importantly, we evaluated the possible correlation between PFO and migraine from the perspective of MRI.

**Figure 2:**
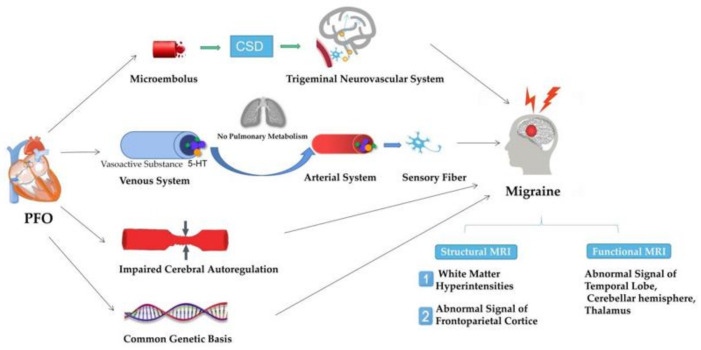
Possible pathophysiological mechanisms and neuroimaging manifestation of PFO and Migraine.

As shown in Figure 2, the possible pathogenesis includes the following four aspects: PFO may trigger CSD through microemboli to trigger the trigeminal neurovascular system, leading to migraine attacks. It is also possible that migraine may be induced by the accumulation of vasoactive substances (such as 5-HT, etc.) caused by PFO in the brain by bypassing the pulmonary metabolism. The dysfunctional autoregulation of intracranial micro-vessels may be involved, and heredity may also play an important role. However, these mechanisms have not been identified to date, and there is a lack of sufficient research to confirm them. In addition, other possible mechanisms need to be explored in the future.

Although most studies point out that PFO is related to migraine, some scholars still argue against the correlation between PFO and migraine, pointing out that the prevalence of PFO in migraine patients is no more common than that in healthy people [81,82]. The reasons for these contradictory findings may be that the inclusion criteria are different, the study populations are inconsistent, and the diagnostic methods of PFO are not sensitive enough.

We collected major studies in the field of migraine neuroimaging (see Figure 2); unfortunately, reliable neuroimaging biomarkers were not identified. We found that neuroimaging research on PFO and migraine is more concentrated in the field of WMH. There are studies supporting the idea that migraine patients with PFO have a higher risk of WMH, and the presence of PFO may affect the location of WMH. Although some people believe that there is no correlation between PFO and WMH [77,83,84,85], we cannot deny the possible shortcomings. For example, a multicenter study [77] in China showed that the presence of PFO did not significantly affect WMH. However, this study only focused on the existence and location of WMH and did not measure the number and total volume of lesions, so it cannot conclusively deny the correlation between RLS and WMH. It is worth noting that the age and gender differences in the subjects also lead to different results. In addition, the impact of RLS on CVR is also controversial. We cannot ignore the study that pointed out that migraine patients with a large RLS show higher BHI [86]. This may be explained by a large number of vasoactive substances bypassing the pulmonary metabolism. However, studies in this area are not sufficient, and important data are still lacking.

In this review, we found that the current research mainly focuses on the white matter of migraine patients with PFO, but few studies focus on the gray matter. More importantly, although there is evidence that PFO is related to abnormal brain functional areas of the temporal lobe, bilateral cerebellar hemisphere, and thalamus, there is a lack of research in the field of fMRI, which suggests that we should pay more attention to these under-researched areas in the next step. It is worth noting that we should not only focus on the FLAIR image of white matter. For example, we could try to use diffusion tensor imaging (DTI) to study the integrity of white matter fiber bundles in migraine patients with PFO to provide new evidence that could provide for further understanding of the connection between PFO and migraine. Furthermore, combined with clinical practice, we need to attempt to solve a problem: that is, whether the emergence of WMH can suggest that patients further screen for PFO. Obviously, solving this problem requires a lot of research data, which also needs to be obtained in the future.

**Figure 3:**
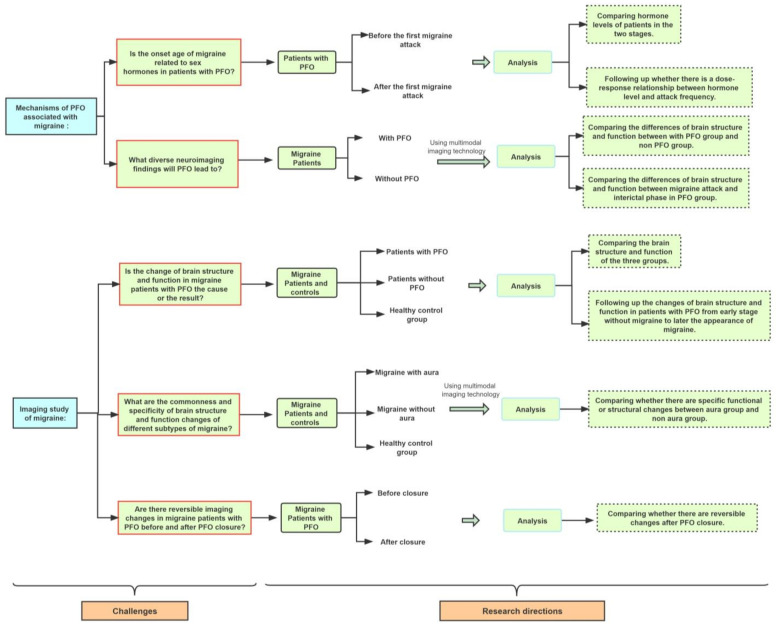
Challenges and future research directions for migraine with PFO.

Figure 3 concludes by showing the existing problems that need to be solved next and the corresponding possible solutions for migraine with PFO. It mainly includes an exploration of the physiological mechanisms of migraine with PFO and the future applications of imaging research. We still need to explore whether hormones are involved in the mechanism of migraine with PFO and how we can establish multimodal imaging technology to study the potential mechanisms of migraine with PFO in the future. However, the changes in brain structure and function in patients with migraine are diverse. Therefore, we should explore whether there are specific bio-imaging changes in different subtypes of migraine, especially in migraine with PFO, the causal relationship between imaging changes and migraine, and whether PFO occlusion can change the brain structure or function of migraine patients with PFO and improve migraine.

If we can solve the problems outlined in Figure 3, this may provide valuable guidance for accurate treatments of migraine with PFO. For example, the current hot issue is whether PFO closure should be performed, but there is no perfect answer to this question. In addition, MRI is still in the transition stage from laboratory to clinic, and it will take some time before it can assist in the diagnosis of each specific clinical patient. Therefore, larger-sample, prospective, multicenter randomized experiments using multimodal magnetic resonance technology are needed in the future.

## 6. Conclusions

Our understanding of the relationship between PFO and migraine continues to evolve, with a large number of studies being conducted on the related mechanisms and neuroimaging manifestations. Four possible mechanisms were found to be involved in the pathophysiological process of PFO-related migraine, including microembolus-triggered CSD, the vasoactive substance hypothesis, impaired cerebral autoregulation, and a common genetic basis. However, there is still uncertainty regarding the pathophysiological mechanisms. Understanding these mechanisms will help to prevent and treat migraine attacks in patients with PFO. In addition, there is much neuroimaging evidence, including structural and functional imaging, to support the possible correlation between PFO and migraine. Specifically, PFO may cause the structure of gray matter to become abnormal and affect the location of WMH, which means that PFO may lead to white matter demyelination and destroy white matter integrity. In addition, PFO may be related to the abnormal performance of functional brain areas involved in driving and integrating migraine attacks. However, the data on functional imaging are still lacking, and further research is needed. Therefore, further study on the pathophysiological mechanisms and multimodal MRI of PFO-related migraine should be encouraged in the future.

## Data Availability

Not applicable.
